# Combination of Plant Growth Regulators, Maltose, and Partial Desiccation Treatment Enhance Somatic Embryogenesis in Selected Malaysian Rice Cultivar

**DOI:** 10.3390/plants8060144

**Published:** 2019-05-30

**Authors:** NG Ja Ming, Suraiya Binte Mostafiz, Nur Syafiqoh Johon, Nur Saliha Abdullah Zulkifli, Alina Wagiran

**Affiliations:** Faculty of Sciences, Universiti Teknologi Malaysia (UTM), Johor Bahru 81310, Malaysia; secretjming@hotmail.com (N.J.M.); bmsuraiya2@live.utm.my (S.B.M.); nursyafiqohjohan@gmail.com (N.S.J.); salihazulkifli@gmail.com (N.S.A.Z.)

**Keywords:** embryogenic callus, 2, 4-Dichlorophenoxyacetic acid, scanning electron microscopy, histology analysis, regeneration, cytokinins, desiccation treatment

## Abstract

The development of efficient tissue culture protocol for somatic embryo would facilitate the genetic modification breeding program. The callus induction and regeneration were studied by using different parameters i.e., auxins, cytokinins, and desiccation treatment. Scanning electron microscopy and histological analysis were performed to identify the embryogenic callus for regeneration. The callus percentage results showed that MS (Murashige and Skoog) basal medium supplemented with 3 mg/L 2, 4-D and 30g/L maltose were the optimal callus induction medium for MR220 (80%) and MR220-CL2 (95%). The morphology of the embryogenic callus was confirmed by the SEM (Scanning Electron Microscopy) (presence of extracellular matrix surface network) and later by histological analysis. Finally, MS media supplemented with 0.5 mg/L NAA (Naphthalene Acetic Acid), 2 mg/L kin, and 1 mg/L BAP were selected as the optimum regeneration media treatment while callus desiccated for 48 h was proved to produce more plantlets in MR220 (60%) and MR220-CL2 (73.33%) compared to control treatment (without desiccation). The protocol presented here showed the necessity for the inclusion of partial desiccation as an important step in the tissue culture protocol of Malaysian *indica* rice genotypes in order to enhance their regeneration potential.

## 1. Introduction

Successful engineered breeding programs have relied on the establishment of efficient regeneration protocols as well as a selection of efficient DNA delivery method, selection, and maintenance of positive transformants [1]. There are a good deal of rice literature [2,3,4] that strategize such protocols to be used in genetic modification studies; however, successful transformation protocols are severely genotype-dependent [2]. Previous research reported that optimal factors for differentiation and regeneration of *indica* rice in vitro were pre-requisites before any genetic transformation programs were employed [3]. Rice callogenesis has been shown to be highly affected by media composition, genotypes, plant growth regulators (PGR), carbohydrates, explants, and adjuvants [4,5,6]. Visual observation using color-base methods has been used to determine embryogenic rice calli; however, these may contribute to the misidentification of features and appearance. Therefore, other convincing tools such as scanning electron microscopy (SEM) and histology analysis are more compatible based on previous literature [7,8]. Hence, determination of embryogenic callus at early callogenesis stages was more effective and reliable in order to minimize the error when selecting the potential of embryogenic callus. Therefore, the present study suggested conducting SEM and histology in the early stages of callogenesis. 

Numerous researches have shown that *indica* rice is less responsive than *japonica* rice to in vitro culture and it still remains a challenging task to achieve a high frequency of somatic embryogenesis with distinct somatic embryo formation. According to previous literature, the establishment of regeneration system of *indica* rice cultivars requires a longer period than *japonica* rice because of the genotypic differences to in vitro culture [3,7]. Until now, there are regeneration protocols for Malaysian rice, however, they are only limited to certain cultivars such as wetland: MR219, MR232, aromatic, MRQ 50, 74, 80, uplands, Kusan, Siam, and Panderas, but not for MR220. The MR220 rice is a commercial cultivar which is well accepted by farmers due to better agronomical characteristics in term of the grain yields and resistance to bacterial leaf blight disease compared to MR219. Following the release of MR220, the MR 220-CL2 cultivar is established to overcome the weedy problem which outgrowths the rice and thus affecting rice yield in Malaysia. The weedy rice has shown the significant effect on the rice yield due to nutritional competition and inability to control weeds using conventional rice herbicides and these led to a newly released herbicide tolerant cultivars, MR220-CL2 at 2010 [9]. In spite of some agricultural advantages, this rice cultivar has not been explored for tissue culture.

Many rice literatures have shown that successful regeneration protocol through somatic embryogenesis (SE) is affected by many factors, such as plant growth regulators, genotypes, or carbohydrates in the induction medium. In case of Malaysian rice, exogenous application of plant growth regulators such as kinetin (Kin), benzyl-amino-purine (BAP), and naphthalene acetic acid (NAA) or thidiazuron (TDZ) could improve regeneration frequency [6]. Moreover, several Malaysian rice cultivars have shown to have poor responses to callus induction, growth, proliferation, and regeneration, such as MRQ72 and MRQ50 [10]. However, the use of suitable plant growth regulars, maltose, amino acids, explant type, and adjuvant materials did increase callusing and regeneration. For examples; MR219 cultivar has been regarded as a recalcitrant cultivar which has shown to be amendable to tissue culture approach [1]. Based on this, it can be observed that Malaysian rice callus induction and regeneration were genotype dependent. Other than that, the inclusion of maltose has been shown to have a promotive effect on regeneration as reported by Zuraida et al. [11]. However, the effect of physical treatment on Malaysian rice is limited for plant regeneration even though it is beneficial for promoting regeneration purposes, whereas another rice genotype has been reported to have partial desiccation treatment [12]. There is no research found on MR220-CL2 and very limited research has been conducted on MR220 regarding the desiccation treatment. Therefore, keeping these issues in view, an attempt was made to enhance the regeneration efficiency in Malaysian rice by exposing the callus to different desiccation durations.

Therefore, the present study was aimed at developing an efficient protocol for callus induction and further plant regeneration of MR220 and MR220-CL2 cultivars by using different 2, 4-D concentrations/basal type, carbohydrate, cytokines, and partial desiccation. There is a need to establish an efficient protocol for Malaysia *indica* rice, especially for MR220 and MR 220-CL2, which can be used in genetic modification studies in the future.

## 2. Results and Discussion

### 2.1. Effect of Basal Medium Type and 2, 4-D Concentration on Callus Induction Percentage

To identify the optimal media composition on callus induction percentage, different 2, 4-D concentrations and two media types were performed. In the present study, callus percentages induced from MR220 and MR220-CL2 cultivars were differed according to 2, 4-D concentrations tested (Table 1). The result showed significant differences when calli in both cultivars were induced on MS media but not for N_6_ media. The callus produced was derived from scutellum after 3 weeks of culture. For MR220 cultivar, the inclusion of 2, 4-D in basal MS media resulted in callus induction percentages ranging from 36.7% to 76.7%. The callus induction percentages were increased with the increment of 2, 4-D concentrations but more than 3 mg/L caused the percentages to decrease. Among the concentration of 2, 4-D tested, the highest percentage of callus induction was on MS medium supplemented with 3 mg/L 2, 4-D (76.7%). Meanwhile, the use of N_6_ media with various concentrations of 2, 4-D showed lower percentages of callus induction compared to MS media. The callus formation was higher when cultured on MS media containing 3 mg/L 2, 4-D (76.7%). The present results showed that the calli of MR220 cultivar were the highest on MS medium compared to N_6_ medium. The new cultivar of MR220 which was CL2 showed the same pattern of callus induction percentage. The highest callus induction percentage of CL2 was observed in MS medium with 3 mg/L 2, 4-D (73.3%), on the other hand, N6 medium with similar 2, 4-D concentration gave lower callus percentage (56.7%). MS media without plant growth regulators showed no callus formation in this study. 

**Figure 1:**
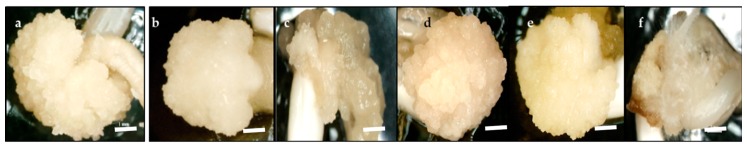
The embryogenic calli (**a**–**c**) MR220 and (**d**–**f**) MR220-CL2 after 3 weeks cultured. (**a**,**d**): Embryogenic calli were compact, nodular structure with light yellowish color at MS medium with 3 mg/L 2, 4-D. (**b**,**e**): Non-embryogenic calli were friable, watery-surface, and loosely held cells on the surface found on N_6_ medium added to 3 mg/L 2, 4-D; (**c**,**f**): Non-embryogenic calli were smaller and loosely held cells on the surface found on N_6_ medium added with 5 mg/L 2, 4-D (Bar = 1 mm).

In the present study, it was observed that the light-yellowish colored calli were produced from scutellum region after 3 weeks on the callus induction media containing 2, 4-D. The morphological observation of calli showed two types of calli; embryogenic and non-embryogenic (Figure 1). The embryogenic calli produced were compact, nodular, dry, big, and yellowish to whitish color (Figure 1a,d) on MS basal medium with 3 mg/L of 2, 4-D, while the non-embryogenic callus with friable, water-surface, and loosely held cells on the surface were observed on N_6_ basal medium with 3 mg/L 2, 4-d (Figure 1b,e) and 5 mg/L of 2, 4-D (Figure 1c,f). 

Based on the morphology of callus, the use of MS media gave rise to embryogenic callus more than N6 media. The calli produced on MS media showed compact, bigger sized, and light-yellowish color (Figure 1a,b) compared to the calli on N6 media which were smaller with water-surface, friable, and yellowish color (Figure 1c,d). Among the treatments, MR220 cultivar showed a higher callus induction percentage (76.7%) compared to MR220-CL2 (73.3%) on MS media containing 3 mg/L 2, 4-D (optimized media). Both cultivars showed a similar tendency of decrease in callus induction when a higher level of 2, 4-D was added to both media. However, no callus was observed on media without any plant growth regulators. In addition, both rice cultivars also responded to a lower calli induction percentages in all treatments with N_6_ media than to those on MS media. According to the results, the percentages of calli induction were significantly different between treatments. In spite of that, the effect of callus induction towards carbohydrate was also investigated in the next step.

### 2.2. Effect of Carbohydrate Sources

**Figure 2:**
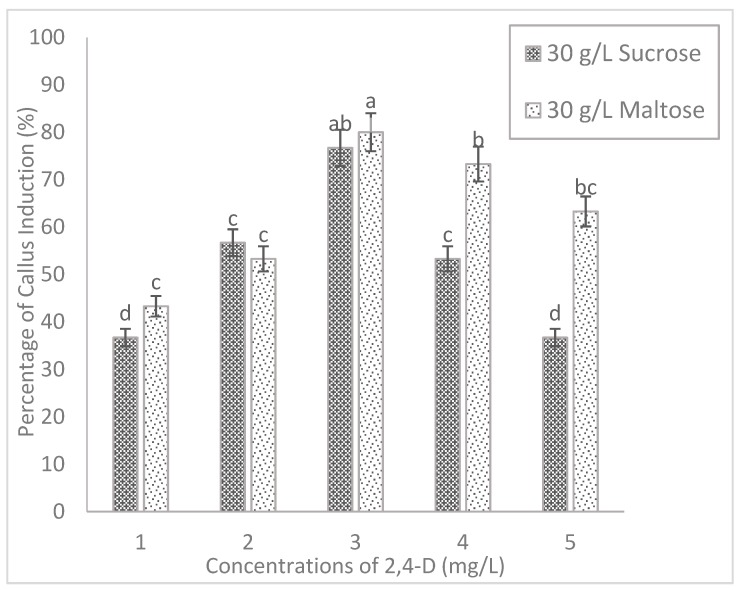
The callus induction of MR220 on MS basal medium supplemented with 30 g/L of sucrose (control) and maltose with different concentrations of 2, 4-D. The sign (I) represents standard error.

**Figure 3:**
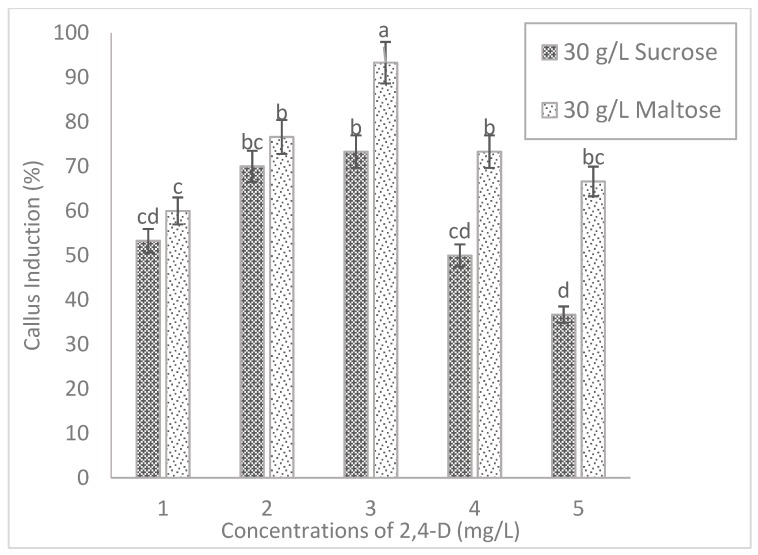
The callus induction of MR220-CL2 on MS basal medium supplemented with 30 g/L of sucrose (control) and maltose with different concentrations of 2, 4-D. The sign (I) represent standard error.

**Figure 4:**
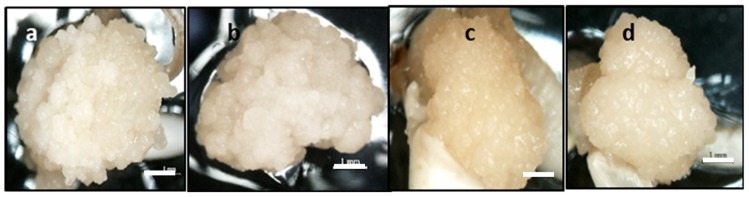
The morphology of calli (**a**,**c**) MR220 and (**b**,**d**) MR220-CL2. (**a**,**b**): Embryogenic calli with compact structure from MS media added with 30 g/L of maltose and 3 mg/L of 2, 4-D; (**c**,**d**): Non-embryogenic with friable and loosely held cell from MS media with 30 g/L of maltose and 1 mg/L 2, 4-D (Bar = 1 mm).

The present study indicated that the inclusion of different carbohydrate sources tends to influence the callus induction significantly in both tested rice cultivars. The callus of MR220 cultured on MS media added to maltose increased significantly from 43.3% (1 mg/L 2, 4-D) to 80% (3 mg/L 2, 4-D) and then decreased to 63.3% (5 mg/L 2, 4-D). Based on Figure 2, the highest callus induction percentages of MR220 (80%) was found on MS media when added with 30 g/L of maltose and 3 mg/L of 2, 4-D. Similarly, the same pattern of callus induction was observed in MR220-CL2. In Figure 3, the highest calli induction percentages in MR220-CL2 was 93.3% when cultured on MS media supplemented with 30 g/L of maltose and 3 mg/L of 2, 4-D and the lowest was observed at 1 mg/L 2, 4-D (60%). Both rice cultivars showed the highest number of calli at MS media with 30 g/L maltose and 3 mg/L 2, 4-D and this treatment was then combined with sorbitol. The calli of MR220 and MR220-CL2 cultivars induced from MS media supplemented with maltose gave rise to embryogenic characteristics showing whitish to light- yellowish color, bigger sized, and compact structure (Figure 4a,b). In contrast, non-embryogenic callus was friable, yellowish, and loosely held cells on the surface (Figure 4c,d).

**Figure 5:**
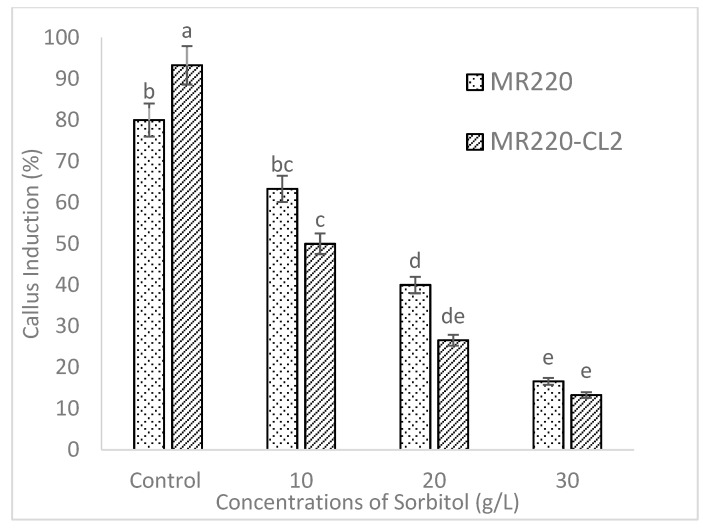
The callus induction of MR220 and MR220-CL2 on MS media with 30 g/L maltose, 3 mg/L of 2, 4-D incorporated with different concentration of sorbitol. I represents standard error.

**Figure 6:**
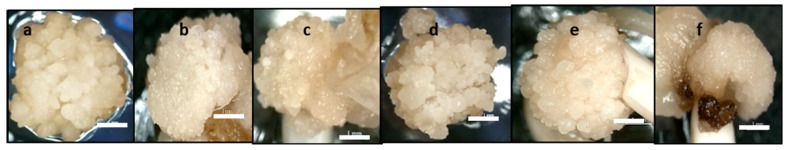
The morphology of calli MR220 (**a**–**c**) and MR220-CL2 (**d**–**f**) on MS media added with 3 mg/L of 2, 4-D, 30 g/L of maltose and different concentration of sorbitol at (**a**,**d**): 10 g/L; (**b**,**e**): 20 g/L and, (**c**,**f**): 30 g/L after 3 weeks cultured (Bar = 1 mm).

According to Figure 5, MS media added with 10 g/L of sorbitol produced a callus induction percentage of 63.3% in MR220 and 50% in MR220-CL2. However, both rice cultivars performed poorly when MS media with 3 mg/L 2, 4-D was supplemented with higher levels of sorbitol (Figure 4). The callus percentages decreased gradually from 63.3% to 16.6% in MR220 cultivar and 50% to 13.3% in MR220-CL2 when 20 g/L and 30 g/L sorbitol, respectively, was used. This showed the inclusion of sorbitol with maltose had a deleterious effect on callus percentage and the results responded to the morphology of the callus as well. Apart from that, the characteristic of embryogenic callus was also observed in the calli of MR220 and MR220-CL2 when cultured on MS media added with 10 g/L sorbitol (Figure 6a,d). However, a higher concentration of sorbitol (20 g/L and 30 g/L) has led to callus browning following reduction in size with yellow color in both cultivars (Figure 6). 

### 2.3. Scanning Electron Microscopy (SEM) and Histology

By using visual observation, the callus can be determined and grouped easily based on morphology and physical properties. However, visual observation may cause misjudgment in the selection of embryogenic cells. It cannot provide the cell composition and structure, which is important information in the production of embryogenic callus. Hence, SEM and histological analysis were used to validate the embryogenic characteristic of the calli in the next step.

**Figure 7:**
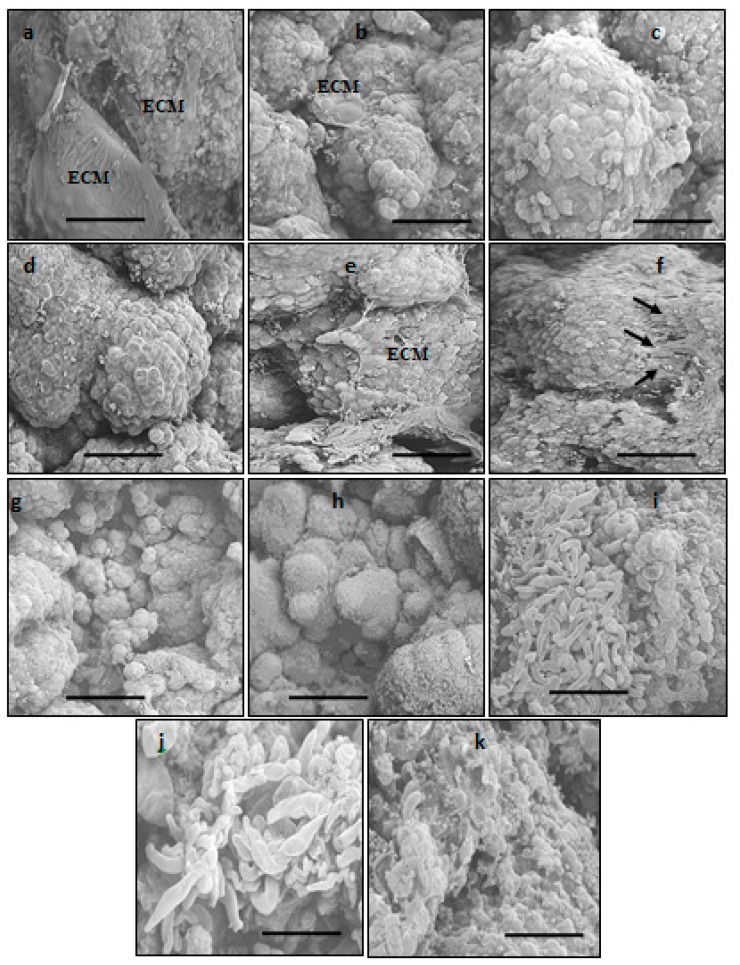
The electron micrograph of MR220 (**a**–**d**,**i**) and MR220-CL2 (**e**–**h**,**j**,**k**). (**a**,**b**,**e**): The embryogenic callus surface covered with thin membranous structure (indicated by extracellular matrix surface (ECM)); (**c**,**d**): Globular, compact structure covered the callus surface; (**f**): Fibrillary structure (indicated by arrows) with packed cells; (**g**): Globular, compact structure within callus cluster; (**h**,**i**): Enlargement detail of non-embryogenic callus surface with long tubular structure; (**j**): Finger-like structure on non-embryogenic callus and (**k**): Unorganized membranous layer on the non-embryogenic callus surface (Bar = 500 mm for (**a–g**,**i**,**j**); 200 mm for (**h**,**k**)).

The two types of calli, both embryogenic and non-embryogenic, of MR220 and MR220-CL2 from the previous experiment were subjected to SEM analysis (Figure 7). The presence of a thin membranous layer which indicated an extracellular matrix surface (ECM) was found on the embryogenic callus surface (Figure 7a,b,e). Fibrils structures have been formed as a network connector between the cells (Figure 7f). The micrograph of SEM showed that a typical embryogenic callus containing globular structure with packed cells which were observed on MR220 (Figure 7c,d). A small organogenic structure adjacent to the callus cluster was observable in MR220-CL2 embryogenic callus (Figure 7g,h) while non-embryogenic calli showed long tubular structures (Figure 7i,j). Moreover, the surface of non-embryogenic callus was unorganized (Figure 7k) which differed with that of embryogenic callus.

**Figure 8:**
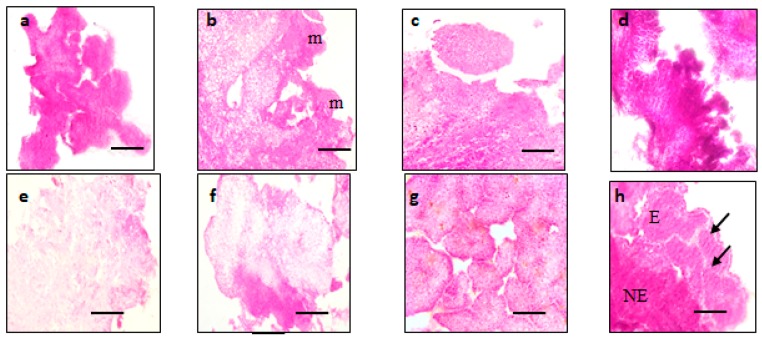
The histology analysis of MR220 (**a**–**e**) and MR220-CL2 (**f**–**h**), (**a**): embryogenic callus; (**b**): densely meristematic cells (indicated as m); (**c**): Pro-embryo with globular shape; (**d**): Cluster of small, globular embryogenic callus unit at the peripheral region; (**e**): the non-embryogenic callus with loose cell and large space; (**f**): Embryogenic callus (E) and non-embryogenic (NE); (**g**): The globular embryogenic callus unit covered with epidermis-like tissue; (**h**): Embryogenic callus units at the peripheral region of the cell under epidermis-like tissue (indicated as arrows) (Bars = 100 mm for (**a**–**h**)).

The SEM result obtained from this study corresponded to the histological analysis (Figure 8). The cross-section of MR220 embryogenic callus with compact cells and nodular appearance were visible under histology analysis (Figure 8a). The external layer of callus was covered with densely meristematic cells and the inner cell layer of callus with highly dense cytoplasm was visible (Figure 8b). Pro-embryo with globular shape was found in Figure 8c and there was a cluster of small pro-embryogenic calli in the peripheral region of the embryogenic cell of MR220 and MR220-CL2 (Figure 8d,g). In addition, part of the MR220-CL2 callus composed of both embryogenic and non-embryogenic cells was indicated in Figure 8f. The calli of MR220-CL2 covered with epidermis-like tissue and parenchymal cells were observed underneath the epidermis tissue (Figure 8h).

### 2.4. Effect of Cytokinin on Regeneration

For regeneration, the embryogenic calli from the previous experiment were cultured on MS media added with 0.5 mg/L NAA and various concentration of kinetin (1–5 mg/L). The effect of kinetin treatments was significantly different towards the percentages (Table 2). The green spot was formed on the embryogenic calli within 14 days after transferring to regeneration media; however, it was found less than 40% on MS media with 2 mg/L kinetin alone in both cultivars. Since the regeneration percentage was low in both cultivars, 2 mg/L of kinetin were combined with different BAP concentrations. Based on Table 2, MS media supplemented with kinetin and BAP were proven to increase regeneration percentages. The optimum regeneration treatment was found in the MS media supplemented with 0.5 mg/L NAA, 2 mg/L kinetin, and 1 mg/L BAP for MR220 (40%) and MR220-CL2 (70%). 

**Figure 9:**
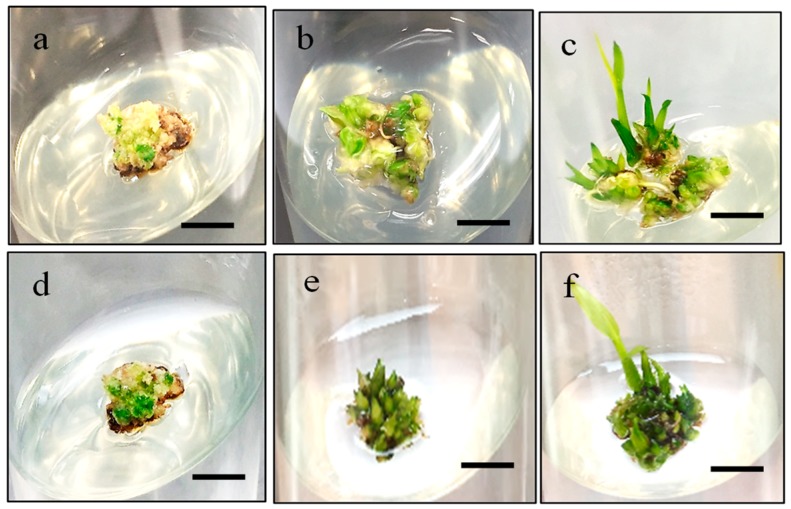
The development of MR220 (**a**–**c**) and MR220-CL2 (**d**–**e**) calli cultured on MS regeneration media supplemented with 0.5 mg/L naphthalene acetic acid (NAA), 2 mg/L kinetin and 1 mg/L benzyl-amino-purine (BAP), from (**a**,**d**) green somatic embryo with adventitious shoot stage, (**b**,**e**) shoot started to emerge and (**c**,**f**) shooting stage (Bar = 1 cm).

The percentages of green spot formation on MR220 and MR220-CL2 were recorded in Table 3. The MS media containing 1 mg/L BAP and 2 mg/L kinetin produced the highest percentage of the green somatic embryo for MR220 (60%) and MR220-CL2 (72.5%). However, the lowest green somatic embryo percentage of MR220 (27.5%) and MR220-CL2 (30%) was observed when added with the highest concentration of BAP (5 mg/L). The green somatic embryo of MR220 and MR220-CL2 were monitored until the development of plantlet. The results revealed that somatic embryo cultured on MS media with kinetin alone (T1–T5) produced fewer plantlets in both cultivars. However, the addition of BAP at 1mg/L showed a profound effect on MR220-CL2 plantlet regeneration (70%) but not for MR220. Figure 9 showed the green somatic embryo from MR220 and MR220-CL2 calli.

### 2.5. Effect of Desiccation Treatment on Regeneration percentage

To evaluate the potentiality of the embryogenic callus response to desiccation, calli from MR220 and MR220-CL2 were exposed to different desiccation treatments (0, 24 h, 48 h, and 72 h) and then cultured on MS added with 0.5 mg/L NAA, 2 mg/L kin, and 1 mg/L BAP. The results revealed that the percentage of plantlets produced was significantly different between the desiccation treatments (Table 4). Based on the result, the green somatic embryo showed non-significant differences in both cultivars among all the treatments (Table 4.). With 48 hours desiccation period (25% water loss), the highest (70%) amount of the green somatic embryo with the adventitious shoot was formed in MR 220 compared to the other treatments. However, the percentage of somatic embryo dropped to 53.3% after 72 h of desiccation (31.7% water loss). Similarly, the green spot formation of MR 220-CL2 ranged from 53.33% to 70%. The percentage of the green somatic embryo of MR 220CL2 was found to be slightly higher than those of green somatic embryo produced in the previous experiment. Both cultivars showed a decrease in percentage of somatic embryo formation after desiccation treatment at 72 h, with the level of water loss more than 25%.

**Figure 10:**
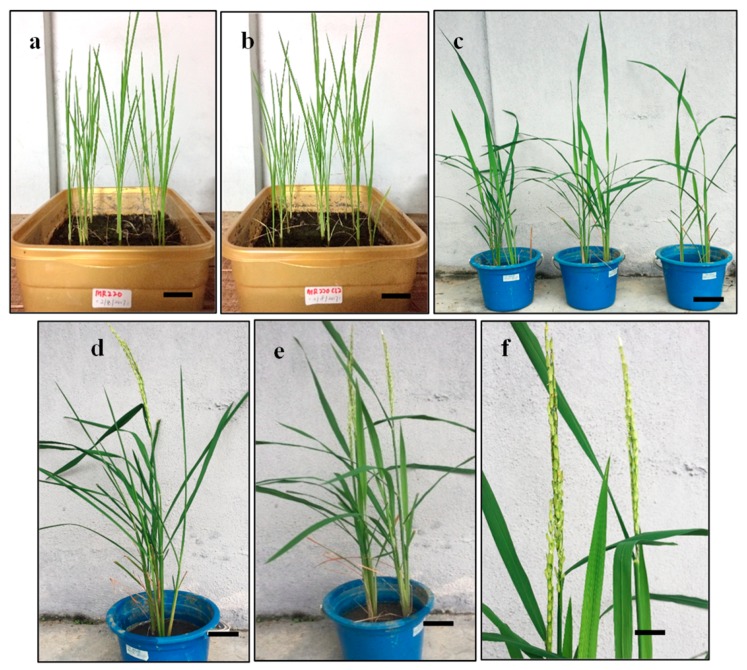
The plantlets of MR220 (**a**,**d**–**f**) and MR220-CL2 (**b**,**c**). (**a**,**b**) The plantlets were growing in the soil after 2 weeks of acclimation. (**c**) The regenerated plants in the pot after 5 weeks. (**d**,**e**) The plant started to flower. (**f**) The grain filling stage of the plant (Bar = 1 cm).

The highest percentages of plantlets of 60% and 73.3% were found in MR220 and MR220-Cl2 respectively at 48 h desiccation treatment. Although the percentage of MR220 plantlets was lower, the production of plantlets was increased from 43% to 60% in this treatment. On the other hand, desiccated calli from 72 h of treatment resulted in plantlet reduction at 20% and 27% for MR220 and MR220-CL2 respectively. This showed that exposure to 72 h desiccation has a deleterious effect on calli. The in vitro plantlets were obtained after 3 weeks cultured in root initiation culture media. Then, these plantlets were transferred into the pot and acclimatized (Figure 10). The roots of in vitro plantlets were washed with autoclaved distilled water before being acclimated into the soil. After 2 weeks of acclimation, the plantlets were healthily growing in soil and started flowering at day 40 for MR220 and 33 for MR 220-CL2.

## 3. Discussions

Embryogenic calli derived from rice scutellum via somatic embryogenesis has been reported to have improved by the optimization of the medium components as well as the type and concentration of plant growth regulators. The results of the callus induced period are in concurrence with the finding of Zuraida et al. [11]. Interaction between genotypes and culture conditions has been reported to influence embryogenic callus formation and quality [13]. The present study showed that the MS medium was found to be superior compared to N6 medium for callus formation and differed in terms of quality and sizes. The callus produced from MS medium was compact, nodular, bigger sized, and light-yellowish color compared to the N_6_ medium, which showed smaller size calli with the watery surface, friable, and yellowish in color. The previous report stated that the calli sizes and appearance were varied among the rice genotypes depending on the selection of basal medium [2]. 

The present study revealed that both MR220 and MR220-CL2 callus induction benefited from the presence of 3 mg/L 2, 4-D and showed different callus percentages. This finding was in agreement with previous research suggesting that the use of 2, 4-D gave successful callus induction in *indica* rice [2,6]. Numerous studies have found out that the addition of 2, 4-D favor callus formation and initiation of the *in vitro* somatic embryogenesis. On the contrary, Zuraida et al. [11] indicated that the addition of higher concentrations of 2, 4-D (10 mg/L) produced callus up to 97% in MR232 cultivar. The 2, 4-D could act as a strong stressor leading to successful somatic embryogenesis and others have found out that 2 and s4-D would regulate somatic embryogenesis through its strong auxinic activity by influencing the endogenous metabolism of other plant growth regulators [14]. 

Regarding the effect of carbon sources, the results in the present study showed that maltose promotes the growth of callus, moreover, the callus induction percentages were higher than the sucrose-containing medium. The calli produced on media added with maltose promoted the growth of embryogenic calli as determined by visual observation. The calli were relatively drier and more compact in morphology. The media supplemented with maltose might control the osmotic potential of the cellular environment of the callus and lead to the production of higher embryogenic calli [2]. The previous study showed that 3% of maltose was found to be the best carbon source for efficient rice callus culture [2]. The present study indicates that the media added with maltose show a low incidence of callus browning than those induced from sucrose. The use of maltose in induction media may help to protect calli from browning by reducing the production of ethylene. Several researchers have summarized that the function of sorbitol in tissue culture acted as a primary carbon source to enhance regeneration percentage of embryogenic callus and during the entire culture period sorbitol might act as an osmotic regulator [12]. However, it was suggested that sorbitol and six-carbon alcohol was commonly regarded as an osmotic agent, which was not metabolized by plant tissue [15,16]. In agreement with this, sorbitol has a deleterious effect on callus formation as shown by a decrease in callusing.

In the present study, the morphological observation of callus showed the embryogenicity and non-embryogenicity based on color [13]. In this study, the embryogenic and non-embryogenic calli of MR220 and MR220-CL2 are discernible based on coloration, structure and size. The result showed that the rice embryogenic calli displayed compact, light yellowish, bigger, and nodular appearance with globular structure when cultured on MS media with optimal plant growth regulator (3 mg/L 2, 4-D), while the non-embryogenic are translucent, watery-surfaced, smaller, and yellowish in color. SEM and histological study were performed to validate the structure of calli and embryogenic competence. According to previous literature, the morphology of embryogenic calli varies in species but similar results have been obtained in cereals and grass [17]. Under the scanning electron microscope, some features common to embryogenic calli, such as the presence of extracellular matrix surface (ECM) in both rice cultivars were observed. The ECM that covered the callus surface was suggested to be an indicator of the embryogenic cell formation that plays a fundamental role in the plant development phase as well as in somatic embryogenesis [6]. The most abundant ECM was observed at the pro-embryo stage and is considered to be a dynamic structure that undergoes continuous change during somatic embryo development. The previous work also stated that calli showed the occurrence of ECM or extracellular fibrillary network, which were restricted to the cell of embryogenic and regenerative potential. Fibrils on the surface of the embryogenic cell and the ECM were arranged as a compact layer of secretion over the cell surface with bridge-like strips between adjacent cells had similar structures with the result obtained in this study [6,7]. In contrast, the non-embryogenic calli covered with long-tubular and unorganized cells were revealed to be the parenchymatous nature of the calli. As reported previously, the ECM was only found on the embryogenic cell or individual pro-embryos, but it was never found on the surface of parenchymatous non-embryogenic callus [3]. 

The SEM results obtained from this study corresponded to the histological analysis. Histological observation of embryogenic calli showed common characteristics to a previous report [7,17] where globular, large protuberance compact structures or small units composed of the packed cell were visible. The peripheral part of the callus had a high density of meristematic cells and the parenchymatic cell was found underneath the epidermis-like tissue. Several types of research had been reported that the parenchymatic cells in the interior part consisted of less nucleus and many vacuoles with external layers of calli which consisted of meristematic cells [17]. The external layers, which consisted of meristematic cells, may lead to the formation of embryogenic cells and somatic embryos. The work was done by Bevitori et al. (2014) [8], who also indicated that the occurrence of epidermis-like layer represented the differentiation processes in progress. The formation of prominent air space in the non-embryogenic cell was due to the differentiation and ultimate death of vacuolated cells in the cell aggregates [18]. Some of the parenchymatic cells and vacuole in the interior of the calli were broken and gave rise to the internal space of the callus. 

Both cytokinins and auxin may interact and promote the development decision toward callogenesis and shoot formation, which plays a role in cell cycle and morphogenic competence in plant growth [6]. The amount of auxin and cytokinin used in the media will affect the development of multiple shoots and hence, decrease the regeneration percentage. The balance between auxin and cytokinins played a major role in the initiation of regeneration induced calli [2,10,11,12]. The unbalanced concentration between auxin and cytokinin has caused a reduction in the regeneration percentages and the multiple shoots proliferation were suppressed [12]. In the present study, the partially desiccation treatment played an effective role to enhance the regeneration, especially for MR220 cultivar. The lower regeneration percentage (10–40%) from the previous experiments tends to increase to 60% from the treatment under desiccation for 48 h. Several types of research of desiccation effect for higher rice regeneration have been studied and the results obtained showed that it was an increment of regeneration up to 2 to 4 fold from desiccated calli [14]. The regenerated result of MR220 obtained in this study was in agreement with the research of Rence et al. [19] who showed that the maximum number of MR220 plantlets produced was from 48 h of desiccation treatment. In addition, the highest number of regeneration was in partially desiccated calli at 48 h [20]. In fact, a higher regeneration is varied across the genotype and depends on the optimum desiccated period. The increment of the desiccated period that causes a loss of more than 20 to 50% water content of the cell would suppress the growth of the embryo, as well as the plantlets regeneration [21]. In this study, the desiccated calli under 72 h of both rice cultivars showed the lowest number of the green somatic embryo and the lowest number of plantlets formation which was in agreement to the previous findings. Moreover, there was only a slightly higher regenerated plantlet of MR220-CL2 from desiccated treatments compared to the control. It may be due to the fact that the degree of water loss differed across different genotypes and the regeneration increased depending on the optimum desiccated period [18,19,22]. 

## 4. Materials and Methods

### 4.1. Plant Materials and Culture Conditions

The mature rice seeds of MR220 cultivar were obtained from the Malaysian Agricultural Research and Development Institute (MARDI), Seberang Prai, Malaysia, while the MR220-CL2 was from Kampung Tengah, Kedah. The dehulled rice seeds were rinsed three times with sterile distilled water prior to immersion in 100% ethanol for one minute. The dehulled seeds were shaken in 100% commercial Clorox (Sodium hypochlorite) with two to three drops of Tween 20 under slight agitation for 30 min. Lastly, the sterilized seeds were rinsed thoroughly several times with sterilized distilled water and blot dried on sterile filter paper before culturing on callus induction media.

### 4.2. Effect of Different Media Types and 2, 4-D on Callus Percentage

To investigate the effect of the media type and 2, 4-D on callus induction, sterilized seeds from various cultivars were cultured on MS [23] or N_6_ [24] which were standardized with 30 g/L sucrose, 2.5 g/L phytagel and different concentration of 2, 4-D (1–5 mg/L). The sterilized seeds were cultured on media without plant growth regulator for the control treatment. 

### 4.3. Effect of Carbohydrates on Callus Percentage

For different carbohydrate tested, 30 g/L of maltose was used while the media added with sucrose acted as a control. In order to investigate the effect of sorbitol, various concentrations of sorbitol (10, 20, & 30 g/L) were added to the above medium. The callus induction medium with optimum plant growth regulator was then added with maltose and sorbitol to evaluate the effect of carbohydrates for callus induction (Table 5). The pH of all media was adjusted to 5.8 prior to autoclave. Each treatment consisted of 10 seeds per plate with 3 replicates and the experiments were repeated three times. All Petri dishes containing sterilized seeds were incubated at 25 ± 2 °C in the dark condition for 6 weeks and sub-cultured at 3 weeks interval. The percentage of callus induction and the morphology of embryogenic and non-embryogenic callus were recorded. Following these, the embryogenic callus was selected for further SEM and histological analysis.

### 4.4. Scanning Electron Microscopy (SEM) and Histological Analysis

Three weeks of embryogenic and non-embryogenic calli were selected and were soaked in FAA (Formalin acetic acid) solution for 24 h. The fixed calli were then dehydrated using a graded ethanol series (70%, 80%, 90% and 100% per hour) and then dried using critical point drying (CPD: Leica). The calli were coated with gold and observed using the scanning electron microscope (Hitachi Tabletop MicroscopeTM3000) at 15 kv. Histology analysis was conducted as described by Vega et al. [17]. The calli were fixed in FAA solution for 24 h and followed by dehydration through a series of ethanol (70%, 80%, 90%, and 100%). The calli were soaked in paraffin wax and then embedded using Tissue Embedding System 2900 (TEC HistoLine Laboratories) in the horizontal orientation using paraffin as embedding medium. The specimen was sectioned at 5 µm and stained using hematoxylin and eosin solution (H&E staining). 

### 4.5. Effect of Cytokinin on Plant Regeneration

The embryogenic calli were selected and cultured on MS basal medium added with 0.5 mg/L NAA, different concentration of kinetin (1–5 mg/L), or in a combination of BAP (1–5 mg/L) separately. All cultures were incubated under 16/8 h (light/dark) photoperiod at 25 ± 2 °C for 4 weeks. Then in vitro shoots whose height reached approximately 2 cm were transferred to half-strength basal MS media without plant growth regulator for root initiation. The percentage of regenerated plantlets and the number of the shoots were recorded at the end of the study.

### 4.6. Effect of Desiccation Treatment on Plant Regeneration

To find out the effect of desiccation on regeneration, callus was subjected to different desiccation treatment. The desiccation treatment was employed by placing callus on sterile filter paper for a period of 24 h, 48 h or 72 h prior to culture on optimal regeneration medium. These desiccated calli were incubated at 25 ± 2 °C under 16 h photoperiod from the white fluorescent lamp (17 µmol/m^2^/s). At the end of the experiment, the percentage of green spot formation and the percentage of plantlets obtained were recorded. 

### 4.7. Statistical Analysis

All mean data were collected and analyzed using statistical software (SPSS 22.0). Each experiment was arranged in a complete randomized design (CRD) with three replicates. Data were analyzed using analyses of variance (ANOVA) and Tukey post hoc test was conducted to determine the significant differences between the treatments at the *P* < 0.05 level of significance. 

## 5. Conclusions

In conclusion, the present study showed that both cultivars (MR220, MR220-CL2) have high calli formation on MS media with 3 mg/L 2, 4-D and maltose. Scanning electron microscopy and histology analysis helped to determine the cell composition and structure of embryogenic callus in both cultivars with respect to the potentiality of regeneration. The effect of cytokinin on MR220 and MR220-CL2 on rice cultivars regeneration was genotype-dependent, while partial-desiccation treatment at 48 h gave a promotive effect on plantlet regeneration percentage (60% for MR220, 73.3% for MR220-Cl2) compared to control. The optimization of desiccation treatment is positively co-related with enhancing the regeneration percentage. The findings of this study could help to improve the quality and yield of MR220 and MR220-Cl2 cultivar through genetic modification studies. 

## Figures and Tables

**Table 1:** Effect of the media type and various 2, 4-D concentrations on the callus percentage of MR220 and MR220-CL2 cultivars.

2, 4-D (mg/L)	MR220		MR220-CL2	
	MS	N_6_	MS	N6
1	36.7 ± 3 a	33.3 ± 8 a	53.3 ± 6 ab	33.3 ± 8 a
2	56.7 ± 3 b	40.0 ± 0 a	70.0 ± 0 bc	40.0 ± 5 a
3	76.7 ± 3 c	53.3 ± 3 a	73.3 ± 3 c	56.7 ± 3 a
4	53.3 ± 3 b	43.3 ± 3 a	50.0 ± 0 a	46.7 ± 6 a
5	36.7 ± 3 a	46.7 ± 6 a	36.7 ± 3 a	43.3 ± 3 a

Value is a means of replicates ± standard error. The value of callus induction percentage followed by different alphabets is significantly different at the 5% probability level according to Tukey’s multiple range test.

**Table 2:** The effect of Kinetin and benzyl-amino-purine (BAP) on the percentage of regenerate plantlet from MR220 and MR220-CL2 cultivars.

Treatment	PGR (mg/L)			Plantlet Regeneration (%)	
	NAA	KIN	BAP	MR220	MR220-CL2
T1	0.5	1	0	20.0 ± 5.77 a	20.0 ± 5.77 ab
T2	0.5	2	0	30.0 ± 5.77 ab	40.0 ± 5.77 b
T3	0.5	3	0	16.6 ± 3.33 a	26.6 ± 3.33 ab
T4	0.5	4	0	16.6 ± 3.33 a	16.6 ± 3.33 a
T5	0.5	5	0	13.3 ± 3.33 a	13.3 ± 3.33 a
T6	0.5	2	1	40 ± 5.77 b	70.0 ± 5.77 c
T7	0.5	2	2	23.3 ± 8.81 ab	46.6 ± 8.81 bc
T8	0.5	2	3	16.6 ± 6.67 a	20.0 ± 5.57 ab
T9	0.5	2	4	13.3 ± 3.33 a	20.0 ± 5.57 ab
T10	0.5	2	5	10.0 ± 0 a	16.6 ± 3.33 a

Values are means of replicates ± standard error. The value of green spot percentage followed by different alphabet is significant differently at the 5% probability level according to Tukey’s multiple range test. (NAA = naphthalene acetic acid).

**Table 3:** The effect of the combination of kinetin and benzyl-amino-purine (BAP) on the green spot formation of MR220 and MR220-CL2 calli.

Treatment		PGR (mg/L)		Green Somatic Embryo (%)	
	NAA	Kinetin	BAP	MR220	MR220-CL2
T5	0.5	0	0	0	0
T6	0.5	2	1	60.0 ± 8.16 c	72.5 ± 9.57 c
T7	0.5	2	2	55.0 ± 5.77 bc	57.5 ± 5 bc
T8	0.5	2	3	40.0 ± 8.16 ab	45.0 ± 5.77 ab
T9	0.5	2	4	35.0 ± 10 a	35.0 ± 5.77 a
T10	0.5	2	5	27.5 ± 5 a	30.0 ± 8.16 a

Value is a means of replicates ± standard error. The value of green spot percentage followed by different alphabet is significant differently at the 5% probability level according to Turkey’s multiple-range test.

**Table 4:** The green somatic embryo formation percentage and number of plantlets regeneration in MR220 and MR 220-CL2 cultivars under different desiccation treatments.

Desiccation Treatment (h)	Green Somatic Embryo (%)		Number of Plantlets Regenerated (%)	
	MR220	MR220-CL2	MR220	MR220-CL2
0 (control)	63.3 ± 3.33	70.0 ± 5.77	43.3 ± 3.11 a	66.6 ± 3.11 ab
24	66.6 ± 3.33	63.3 ± 3.33	50.0 ± 3.11 ab	70.0 ± 3.11 b
48	70.0 ± 5.77	66.6 ± 3.33	60.0 ± 3.11 ab	73.3 ± 3.11 b
72	53.3 ± 3.33	53.3 ± 3.33	40.0 ± 3.11 a	46.3 ± 3.11 a

Value is a means of replicates ± standard error. The value of green spot percentage followed by different alphabet is significant differently at the 5% probability level according to Turkey’s multiple-range test.

**Table 5:** The carbohydrate concentration used in callus induction.

Carbohydrate (g/L)	MS/N_6_ Media	MS Media	MS
Sucrose (control)	30	0	0
Maltose	0	30	30
Sorbitol	0	0	10, 20, 30
